# Hepatotoxicity and efficacy associated with first- and new-generation EGFR-TKIs in patients with NSCLC: a systematic review and meta-analysis

**DOI:** 10.1186/s12885-025-15330-2

**Published:** 2025-12-29

**Authors:** Zhe Wang, Jipeng Meng, Guanlin Liu, Yidan Wang, Yi Li, Chengrui Zhang, Yong Liu, Guoxiang Sun

**Affiliations:** 1 School of Pharmacy, Shenyang Pharmaceutical University, 103 Wenhua Road, Shenyang, Shenhe District 110016 China; 2 Institute of Catalysis for Energy and Environment, College of Chemistry and Chemical Engineering, Shenyang Normal University, Shenyang, 110034 China; 3 School of Chemical Engineering, Ocean and Life Sciences, Dalian University of Technology, Panjin, 124221 China

**Keywords:** EGFR-TKIs, NSCLC, Hepatotoxicity, Meta-analysis

## Abstract

**Background:**

While hepatotoxicity has been widely reported with epidermal growth factor receptor tyrosine kinase inhibitors (EGFR-TKIs), the comparative risk among them remains unclear. This study aimed to directly compare the relative risk (RR) of hepatotoxicity between new-generation (afatinib, osimertinib, dacomitinib) and first-generation (gefitinib, erlotinib) EGFR-TKIs in non-small-cell lung cancer (NSCLC) and to evaluate their overall risk-benefit profile.

**Methods:**

PubMed, Embase, Cochrane library databases and clinicaltrials.gov were searched for trials up to September 2025. A study protocol was registered in PROSPERO: CRD42023457906. Among the 5371 records identified, 6 studies finally fulfilled the established criteria. Data extracted for each study included study characteristics, baseline patient information, interventions and data on all-grades alanine aminotransferase (ALT), aspartate aminotransferase (AST), and total bilirubin (TB) elevation, overall survival (OS), progression-free survival (PFS) and objective response rate (ORR). RR, hazard ratio (HR) and 95% confidence interval (CI) were calculated using the inverse variance method.

**Results:**

Six trials involving 2528 patients were analyzed. Decreased risks of hepatotoxicity due to the elevation of AST and ALT were observed for each new-generation EGFR-TKI. The pooled RRs of all-grades ALT, AST and TB elevation were 0.36 (95% CI 0.24–0.52, *P* < 0.001), 0.44 (95% CI 0.36–0.54, *P* < 0.001) and 0.83 (95% CI 0.50–1.39, *P* = 0.48), respectively. New-generation TKIs did achieved benefit in PFS (HR 0.65, 95% CI 0.50–0.83, *P* < 0.0001) and ORR (RR 1.14, 95% CI 1.00-1.29, *P* = 0.04). The OS of patients with new-generation TKI treatment was extended (afatinib, HR 0.73, 95% CI 0.58–0.92, *P* = 0.008 and osimertinib, HR 0.71, 95% CI 0.53–0.95, *P* = 0.02), except dacomitinib (HR 0.97, 95% CI 0.72–1.29, *P* = 0.81).

**Conclusions:**

New-generation EGFR-TKIs (afatinib, osimertinib, and dacomitinib) demonstrate a superior efficacy and safety profile, with a significantly lower risk of hepatotoxicity, compared to gefitinib and erlotinib.

**Graphical Abstract:**

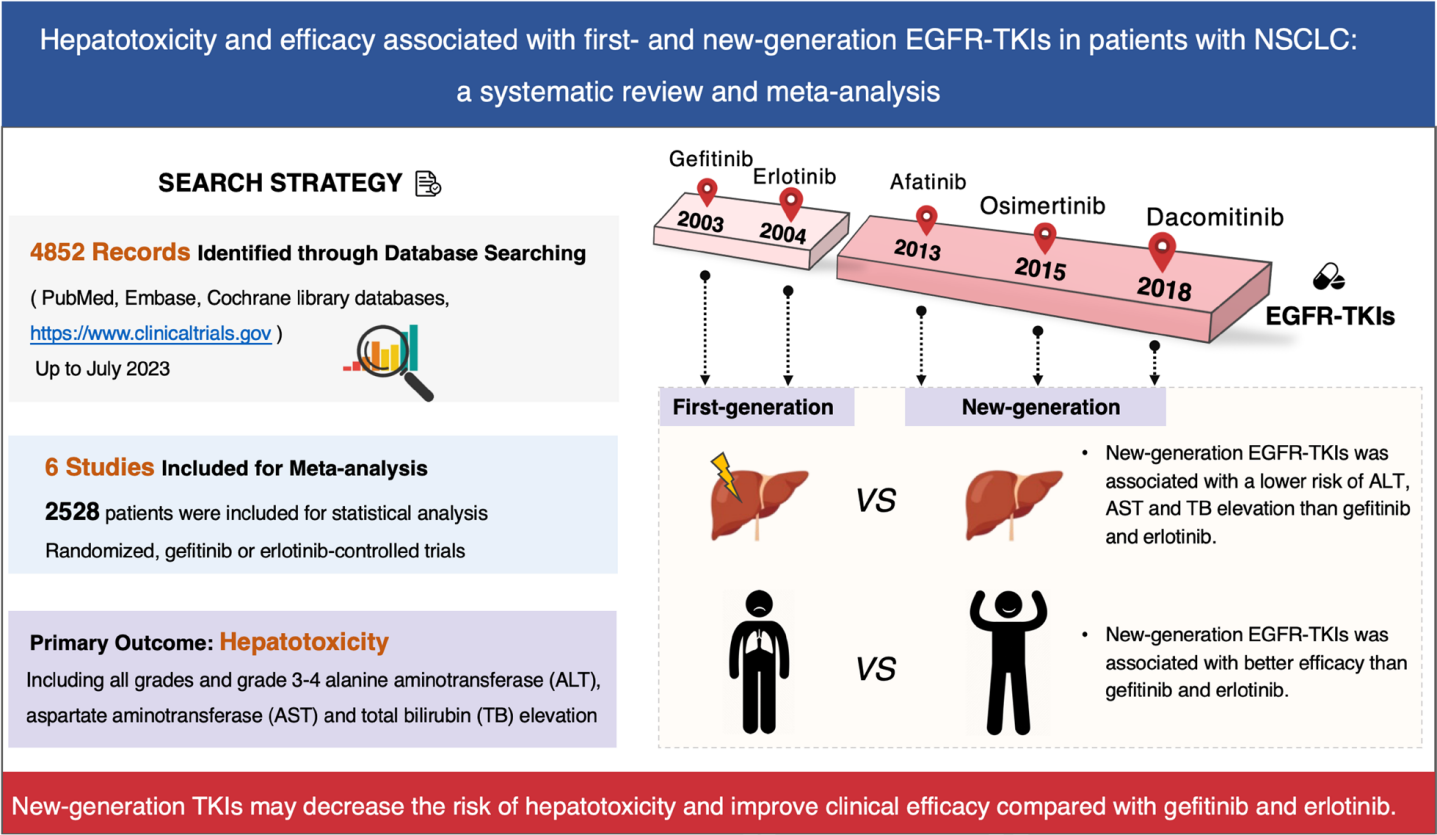

**Supplementary Information:**

The online version contains supplementary material available at 10.1186/s12885-025-15330-2.

## Introduction

Lung cancer remains the leading cause of cancer death worldwide [1], and non-small-cell lung cancer (NSCLC) accounts for approximately 85% of cases, with up to 50% of Asian patients harboring epidermal growth factor receptor (EGFR) mutations [2]. Gefitinib, erlotinib, afatinib, dacomitinib and osimertinib are tyrosine kinase inhibitors (TKIs) approved by Food and Drug Administration (FDA) for the first-line treatment of metastatic NSCLC. While all target the classic exon 19 deletion and exon 21 L858R mutations, osimertinib is uniquely approved for tumors with the T790M resistance mutation, a primary reason for its widespread use [3]. These targeted therapies and emerging combined treatment regimens have significantly improved the survival rate of patients [4–7].

Drug-induced hepatotoxicity constitutes a major challenge in both drug development and clinical therapy, with nearly 50% of marketed oral drugs being associated with hepatotoxicity [8]. This concern extends to EGFR-TKIs. Despite first-generation agents (gefitinib and erlotinib) being standard first-line treatments for EGFR-mutant NSCLC, they have been consistently linked to severe, and sometimes fatal, hepatotoxicity [9–13]. Similarly, new-generation EGFR-TKIs carry hepatotoxic risks; afatinib therapy commonly leads to elevated serum aminotransferase levels [14, 15], and its official label includes warnings of fatal hepatic events [16]. Furthermore, cases of liver injury have been documented with osimertinib and dacomitinib [17–22].

While previous meta-analyses have evaluated the overall efficacy and safety profiles of EGFR-TKIs [23–27], a dedicated and direct comparison of their hepatotoxic risks is lacking. To our knowledge, only one such study has focused on hepatotoxicity, and it did not perform head-to-head comparisons between the different generations of drugs [28]. In order to obtain more intuitive conclusions and provide references for clinical administration, it is necessary to conduct a systematic meta-analysis of the relative risk of hepatotoxicity associated with each EGFR-TKIs.

This meta-analysis was conducted to directly compare the risk of developing all-grades hepatotoxicity associated with new-generation EGFR-TKIs (afatinib, osimertinib and dacomitinib) vs. first-generation EGFR-TKIs (gefitinib and erlotinib) in patients with NSCLC. Stratification by EGFR-TKIs treatment was performed to provide drug-specific risk assessment. Overall survival (OS), progression-free survival (PFS) and objective response rate (ORR) were also analyzed to provide global risk-benefit evaluation.

## Materials and methods

### Definition of the outcome

The primary outcome of this analysis was hepatotoxicity, including the risk of all-grades alanine aminotransferase (ALT), aspartate aminotransferase (AST) and total bilirubin (TB) elevation. Hepatotoxicity events were defined as the National Cancer Institute Common Terminology Criteria for Adverse Events, version 3.0 or 4.0 (CTCAE: http://ctep.cancer.gov). Secondary outcomes were OS, PFS and ORR.

### Search strategy and study selection

We systematically searched PubMed, Embase, Cochrane Library and ClinicalTrials.gov databases up to September 2025, following a literature search strategy by combining entry terms of the words ‘‘gefitinib, erlotinib, afatinib, osimertinib, and dacomitinib” with ‘‘non-small-cell lung cancer” (Method in the Supplement). The inclusion and exclusion criteria have been previously published in the International Prospective Register of Systematic Reviews (PROSPERO: CRD42023457906). In this analysis, only randomized phase 2 or phase 3 clinical trials that compared a new-generation EGFR-TKIs (afatinib, osimertinib, and dacomitinib) with first-generation EGFR-TKIs (gefitinib and erlotinib) were included. If multiple publications of the same trial were identified, only the most recent and informative publication was selected. Study quality was assessed by Jadad scale [29].

### Data extraction

Data collection was performed by two independent investigators, and any disagreements were resolved through a third investigator. We extracted the following information from each selected study: author’s name, year of publication, trial phase, number of patients, drugs, dosage, frequency, type of cancer and stage, median age, gender, and data on ALT, AST, TB, PFS, OS, and ORR. Some of these data were found at clinical trials.gov to ensure data integrity.

### Statistical analysis

RevMan version 5.3 (Cochrane Library, Oxford, UK) was used to perform statistical analyses. The relative risk (RR) and 95% confidence interval (CI) were calculated using the inverse variance method to assess the risk of all-grades ALT, AST, TB elevation, and ORR of EGFR-TKIs. The hazard ratio (HR) was calculated to assess the PFS and OS. The heterogeneity among trials was examined by I^2^ statistic. When substantial heterogeneity was observed (I^2^>50%), the random effect model was used. Otherwise, the fixed effect model was used. A P-value of < 0.05 was considered to be statistically significant. Additionally, funnel plot asymmetry was used to assess the publication bias of the enrolled studies.

## Results

### Study selection

**Fig. 1:**
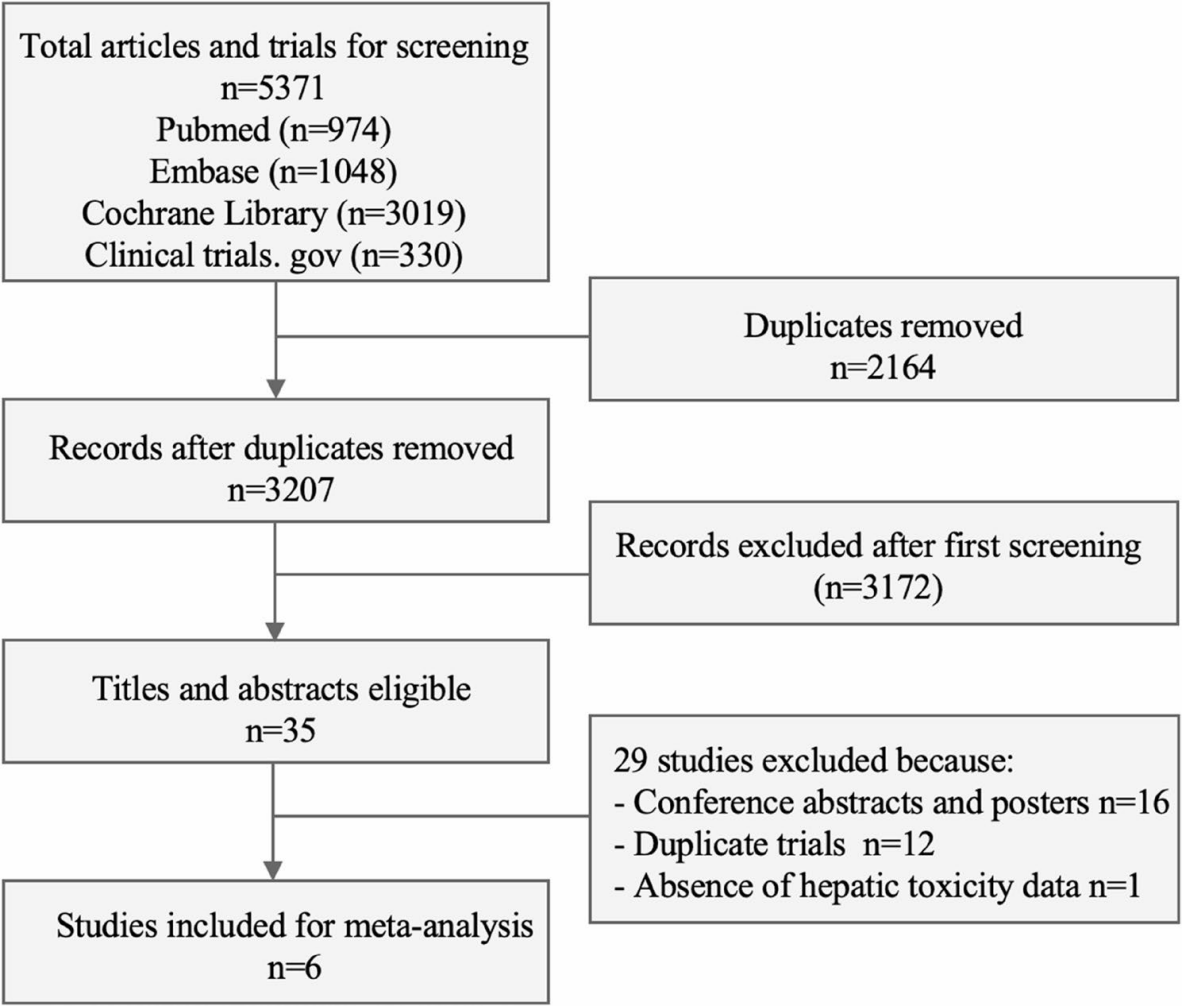
Studies eligible for inclusion in the meta-analysis

The process of selecting eligible studies is shown in Fig. 1. A total of 5371 records was identified in the initial search (PubMed 974, Embase 1048, Cochrane library 3019, and clinical trials.gov 330), and 2164 repetitive articles were deleted. After reading titles and abstracts, 35 eligible articles and abstracts were selected. Among them, 16 studies were conference abstracts and posters, 12 studies were identified as duplicate trials, and one was excluded due to lack of hepatic toxicity data [30]. Finally, a total of 6 studies with 2528 patients were included in the meta-analysis [31–36].

### Characteristics of the studies and quality assessment

All included studies in this study were randomized, gefitinib- or erlotinib-controlled trials published from 2012 to 2021. TKIs included in the analysis were afatinib (*n* = 1), osimertinib (*n* = 2), and dacomitinib (*n* = 3). All patients were diagnosed with NSCLC; male patients made up 48.7% and patient age ranged from 24 to 93 years with a median of 62 years. Other characteristics and Jadad score of the included studies are summarized in Table 1. Funnel plots for the primary and secondary outcomes were largely symmetrical (Figure in the Supplement), which suggested that publication bias was unlikely among the studies.

**Table 1:** Characteristics of included studies and quality assessment

Authors (year)	Trial register	Phase	Trial design	Treatment arm	Number of patients	Population	Median age (years)	Male, *n*(%)	Jadad score
Keunchil Park (2016)	NCT01466660	Ⅱ	RCT	afatinib 40 mg/day	160	treatment-naive stage IIIB or IV NSCLC and one EGFR mutation	63(30–86)	69(43%)	3
				gefitinib 250 mg/day	159		63(32–89)	53(33%)	
Jean-Charles Soria (2017)	NCT02296125	Ⅲ	RCT	osimertinib 80 mg/day	279	EGFR mutation-positive advanced NSCLC	64(26–85)	101(36%)	3
				gefitinib or erlotinib 250 mg/day, 150/100 mg/day	277		64(35–93)	105(38%)	
Ying Cheng (2021)	NCT02296125	Ⅲ	RCT	osimertinib 80 mg/day	71	EGFR mutation-positive advanced NSCLC	60 (29–80)	28(39%)	3
				gefitinib or erlotinib 250 mg/day, 150/100 mg/day	65		61 (32–82)	19(29%)	
Suresh S Ramalingam (2012)	NCT00769067	Ⅱ	RCT	dacomitinib 45 mg/day	93	advanced NSCLC	60 (24–82)	55(59%)	2
				erlotinib 150 mg/day	94		62 (27–85)	56(60%)	
Suresh S Ramalingam (2014)	NCT01360554	Ⅲ	RCT	dacomitinib 45 mg/day	439	advanced NSCLC	64(32–86)	288(66%)	5
				erlotinib 150 mg/day	439		62 (34–88)	277(63%)	
Yi-Long Wu (2017)	NCT01774721	Ⅲ	RCT	dacomitinib 45 mg/day	227	newly diagnosed advanced NSCLC and one EGFR mutation	62(53–68)	81(36%)	3
				gefitinib 250 mg/day	225		61(54–68)	100(44%)	

### Primary and secondary outcomes

The fixed or random effects model RR or HR for the hepatotoxicity (ALT, AST and TB elevation), PFS, OS, and ORR were summarized in Tables 2 and 3, respectively. The description of heterogeneity is also provided.

**Table 2:** RR of liver toxicity associated with EGFR-TKIs in the treatment of NSCLC

TKI	RR (95% CI)	I^2^ statistic	*P* value
All-grades ALT elevation			
Overall	0.36 [0.24, 0.52]	55%	0.000
Afatinib	0.45 [0.28, 0.73]	NA	0.000
Osimertinib	0.22 [0.15, 0.34]	0%	0.000
Dacomitinib	0.48 [0.36, 0.64]	0%	0.000
All grade AST elevation			
Overall	0.44 [0.36, 0.54]	0%	0.000
Afatinib	0.42 [0.24, 0.72]	NA	0.002
Osimertinib	0.37 [0.26, 0.53]	0%	0.000
Dacomitinib	0.51 [0.38, 0.68]	0%	0.000
All grade total bilirubin elevation			
Overall	0.83 [0.50, 1.39]	49%	0.48
Afatinib	2.98 [0.12, 72.64]	NA	0.50
Osimertinib	0.61 [0.18, 2.07]	NA	0.43
Dacomitinib	0.86 [0.48, 1.52]	80%	0.60

**Table 3:** RR or HR of progression-free survival, overall survival and overall response rate associated with EGFR-TKIs in the treatment of NSCLC

TKI	RR or HR (95% CI)	I^2^ statistic	*P* value
Progression-free survival			
Overall	0.65 [0.50, 0.83]	84%	0.000
Afatinib	0.73 [0.57, 0.95]	NA	0.02
Osimertinib	0.48 [0.40, 0.58]	0%	0.000
Dacomitinib	0.72 [0.52, 1.00]	84%	0.05
Overall survival			
Overall	0.82 [0.66, 1.02]	68%	0.08
Afatinib	0.73 [0.58, 0.92]	NA	0.008
Osimertinib	0.71 [0.53, 0.95]	17%	0.02
Dacomitinib	0.97 [0.72, 1.29]	58%	0.81
Overall response rate			
Overall	1.14 [1.00, 1.29]	59%	0.04
Afatinib	1.25 [1.05, 1.48]	NA	0.01
Osimertinib	1.05 [0.96, 1.15]	NA	0.24
Dacomitinib	1.36 [0.88, 2.10]	70%	0.16

### ALT elevation

**Fig. 2:**
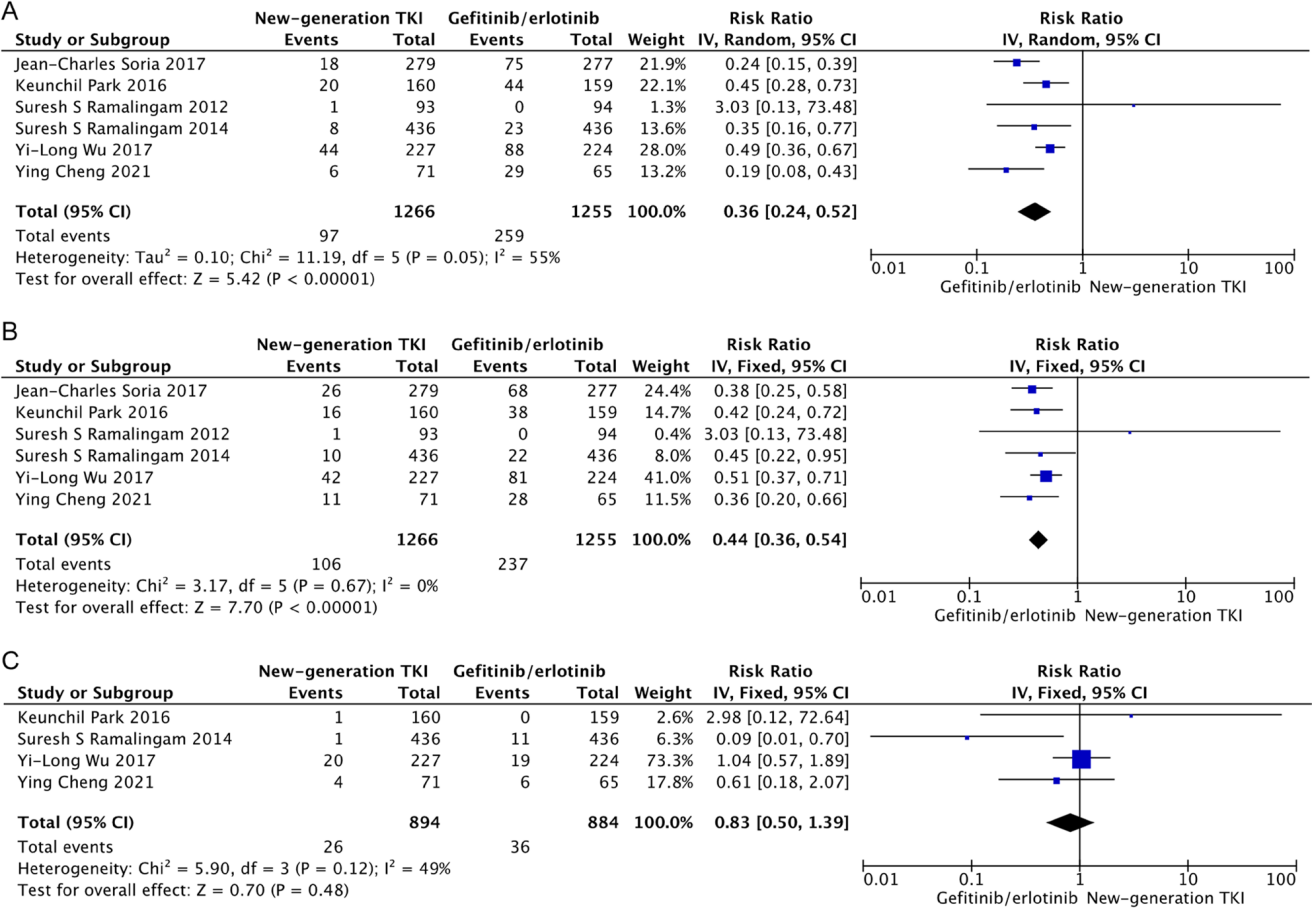
Pooled analysis of all-grades ALT (**A**), AST (**B**) and total bilirubin (**C**) elevation

All-grades ALT elevation occurred in 7.7% of patients (97 of 1266) treated with new-generation TKIs vs. 20.6% of patients (259 of 1255) treated with gefitinib or erlotinib. The risk of developing all-grades of ALT elevation was significantly lower with the use of new-generation TKIs than with gefitinib or erlotinib (RR = 0.36, 95% CI, 0.24–0.52; *P* < 0.001) (Fig. 2A). When stratified by type of drug, afatinib (RR = 0.45, 95% CI, 0.28–0.73; *P* < 0.001), osimertinib (RR = 0.22, 95% CI, 0.15–0.34; *P* < 0.001), and dacomitinib (RR = 0.48, 95% CI, 0.36–0.64; *P* < 0.001) decreased the RR for all-grades ALT elevation.

### AST elevation

All-grades AST elevation was observed in 8.4% of patients (106 of 1266) treated with new-generation TKIs vs. 18.9% of patients (237 of 1255) treated with gefitinib or erlotinib. And the risk of developing all-grades AST elevation was significantly lower with the treatment of new-generation TKIs compared to gefitinib or erlotinib (RR = 0.44, 95% CI, 0.36–0.54; *P* < 0.001) (Fig. 2B). Subgroup analysis indicated that the difference was statistically significant for afatinib (RR = 0.42, 95% CI, 0.24–0.72; *P* = 0.002), osimertinib (RR = 0.37, 95% CI, 0.26–0.53; *P* < 0.001), and dacomitinib (RR = 0.51, 95% CI, 0.38–0.68; *P* < 0.001).

### TB elevation

As shown in Fig. 2C, all-grades TB elevation occurred in 2.9% of patients (26 of 894) treated with new-generation TKIs vs. 4.1% of patients (36 of 884) treated with gefitinib or erlotinib. And there was no statistical difference in all-grades TB elevation between new-generation and first-generation TKIs (RR = 0.83, 95% CI, 0.50–1.39; *P* = 0.48). Stratification by treatment did not change the results (afatinib, RR = 2.98, 95% CI, 0.12–72.64; *P* = 0.50; osimertinib, RR = 0.61, 95% CI, 0.18–2.07; *P* = 0.43; dacomitinib, RR = 0.86, 95% CI, 0.48–1.52; *P* = 0.60).

### Progression-free survival

**Fig. 3:**
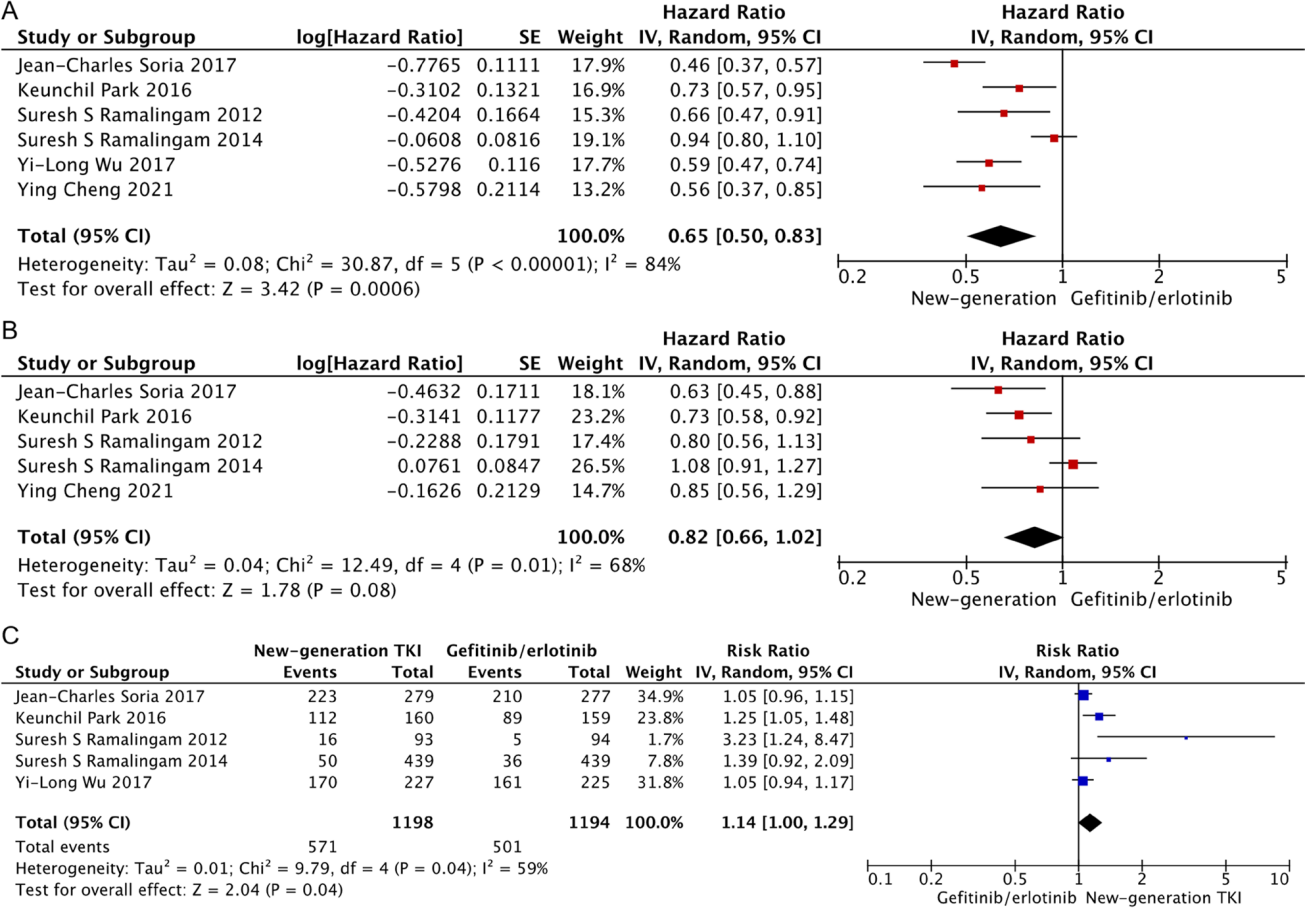
Pooled analysis progression-free survival (**A**), overall survival (**B**) and overall response rate (**C**)

Pooling the PFS data showed that new-generation EGFR-TKIs significantly prolong the PFS (HR = 0.65, 95% CI, 0.50–0.83; *P* < 0.0001) compared with the first-generation TKIs (Fig. 3A). Similar results were observed for each TKI when subgroup analysis was performed (afatinib, HR = 0.73, 95% CI, 0.57–0.95; *P* = 0.02; osimertinib, HR = 0.48, 95% CI, 0.40–0.58; *P* < 0.0001; dacomitinib, HR = 0.72, 95% CI, 0.52–1.00.52.00; *P* = 0.05).

### Overall survival

As shown in Fig. 3B, there was no statistical difference in OS between new- and first-generation EGFR-TKIs (HR = 0.82, 95% CI, 0.66–1.02; *P* = 0.08), with statistical significance between-study heterogeneity (I^2^ = 68%). Subgroup analysis showed an improvement in OS for patients treated with afatinib (HR = 0.73, 95% CI, 0.58–0.92; *P* = 0.008) and osimertinib (HR = 0.71, 95% CI, 0.53–0.95; *P* = 0.02), but not dacomitinib (HR = 0.97, 95% CI, 0.72–1.29; *P* = 0.81). ARCHER 1009 trial is a source of heterogeneity. The 1-way sensitivity analysis also indicated that removing the ARCHER 1009 trial (NCT01360554) significantly affected the overall results (HR = 0.73, 95% CI, 0.63–0.86; *P* < 0.0001).

### Objective response rate

As shown in Fig. 3C, 47.7% (571 out of 1198) patients treated with new-generation TKIs showed complete or partial response against 41.9% (501 out of 1194) of patients treated with gefitinib or erlotinib. And the pooling ORR data achieved significant advantage in the new-generation TKIs (RR = 1.14, 95% CI, 1.00–1.29.00.29; *P* = 0.04). Similar results were observed for each TKI, especially for afatinib (afatinib, RR = 1.25, 95% CI, 1.05–1.48; *P* = 0.01; osimertinib, RR = 1.05, 95% CI, 0.96–1.15; *P* = 0.24; dacomitinib, RR = 1.36, 95% CI, 0.88–2.01; *P* = 0.16).

## Discussion

As of September 2025, there are 93 FDA-approved TKIs for human use [37], among which six carry black box warnings for liver toxicity (sunitinib, lapatinib, pazopanib, regorafenib, ponatinib, and pexidartinib). Given that hepatotoxicity is a predominant side effect of TKIs therapy, most of these drugs require liver function monitoring. A previously published meta-analysis found that patients receiving TKIs had a 4-fold higher risk of experiencing high-grade hepatic adverse events compared to those receiving placebo [38]. A recent network meta-analysis indicated that ALT and AST elevation were more frequent in patients treated with gefitinib and erlotinib than in those treated with afatinib and dacomitinib [28], but it did not make a direct comparison.

To the best of our knowledge, this is the first study performing a meta-analysis to directly and separately compare risks of ALT, AST and TB elevation among new-generation and first-generation EGFR-TKIs in patients with NSCLC. To assess global risk-benefit, survival efficacy and objective response were also analyzed. Based on direct evidence, we were able to demonstrate that treatment with the new-generation EGFR-TKIs (afatinib, osimertinib, and dacomitinib) was associated with a lower risk of hepatotoxicity and better efficacy than gefitinib and erlotinib.

The mechanisms of TKI-induced hepatotoxicity are not fully elucidated but are thought to involve reactive metabolites (RMs) [39]. Previous studies have demonstrated that the cytochrome P450-mediated oxidative metabolism of gefitinib and erlotinib generates RMs, such as quinone-imine, ketene, oxirene, and epoxides [40–43]. As electrophiles, these RMs can covalently bind to nucleophilic proteins and DNA, leading to cellular damage and death. The immune system has also been implicated in gefitinib-induced liver injury [44, 45]. Another mechanism involves UGT1A1 inhibition. As bilirubin is metabolized by UGT1A1, its inhibition by gefitinib, erlotinib, and osimertinib may explain some hepatic adverse effects [46, 47]. In the current meta-analysis, afatinib, osimertinib, and dacomitinib were significantly associated with a lower risk of hepatotoxicity due to the elevation of AST and ALT. There was however a lack of significant effect on total bilirubin elevation. This may indicate that the hepatotoxicity of EGFR-TKIs is primarily hepatocellular rather than cholestatic [48]. Overall, the exact mechanisms of TKI-associated hepatotoxicity warrant further investigation.

There were several limitations in the current analysis. Firstly, only six head-to-head randomized trials met eligibility, and none were prospectively powered for hepatotoxicity. Secondly, many studies lack reports of hepatotoxic events, and excluding them may introduce bias in the analysis. Thirdly, patient-level variability (EGFR mutation spectrum, line of therapy, prior treatments, dosing, regional enrollment) likely contributed to between-study heterogeneity. Finally, the limited and inconsistent reporting of alkaline phosphatase (ALP) and gamma-glutamyl transferase (γ-GT) in the included RCTs precluded their inclusion, potentially reducing the accuracy of our hepatotoxicity assessment.

In conclusion, afatinib, osimertinib, and dacomitinib demonstrated superior efficacy and a significantly lower risk of hepatotoxicity compared to gefitinib and erlotinib. Our findings strongly support the use of these new-generation EGFR-TKIs in patients with NSCLC from a hepatotoxicity perspective.

## Supplementary Information

Supplementary Material 1.

Supplementary Material 2.

## Data Availability

The datasets used and analysed during the current study are available from Zhe Wang (Email: wangzhe108@sina.cn).
