# Supplementary material for: Hepatotoxicity and efficacy associated with first- and new-generation EGFR-TKIs in patients with NSCLC: a systematic review and meta-analysis
Source: BMC Cancer. 2025 Dec 29;25:1909. doi: 10.1186/s12885-025-15330-2 (PMC12751619; doi:10.1186/s12885-025-15330-2)
Supplement: Supplementary file 1 — Supplementary Material 1. [file 12885_2025_15330_MOESM1_ESM.docx]

**Supplementary Online Content**

Method: Search strategy.

Figure: Evaluation of publications bias.

**Method: Search strategy (up to September 2025)**

**A. Gefitinib**

**a) Database: PubMed (354)**

Search details: search ((((gefitinib[MeSH Terms]) OR (N-(3-Chloro-4-fluorophenyl)-7-methoxy-6-(3-(4-morpholinyl)propoxy)-4-quinazolinamide[Title/Abstract])) OR (Iressa[Title/Abstract])) OR (ZD1839[Title/Abstract])) AND (non-small cell lung cancer[MeSH Terms]) Filters: Clinical Trial

**b) Database: Embase (312)**

Search details were same to Pubmed.

**c) Database: The Cochrane Library (847)**

Search details:

#1 (gefitinib):ti,ab,kw OR (N-(3-Chloro-4-fluorophenyl)-7-methoxy-6-(3-(4-morpholinyl)propoxy)-4-quinazolinamide):ti,ab,kw OR (Iressa):ti,ab,kw OR (ZD1839):ti,ab,kw (Word variations have been searched)

#2 (non-small cell lung cancer):ti,ab,kw

#3 #1 and #2

**B. Erlotinib**

**a) Database: PubMed (523)**

Search details: search ((((((((((((((((((((erlotinib[MeSH Terms]) OR (Hydrochloride, Erlotinib[Title/Abstract])) OR (Erlotinib HCl[Title/Abstract])) OR (HCl, Erlotinib[Title/Abstract])) OR (OSI-774[Title/Abstract])) OR (OSI 774[Title/Abstract])) OR (OSI774[Title/Abstract])) OR (CP 358774[Title/Abstract])) OR (358774, CP[Title/Abstract])) OR (CP 358,774[Title/Abstract])) OR (358,774, CP[Title/Abstract])) OR (CP-358,774[Title/Abstract])) OR (CP358,774[Title/Abstract])) OR (CP-358774[Title/Abstract])) OR (CP358774[Title/Abstract])) OR (11C-erlotinib[Title/Abstract])) OR (11C erlotinib[Title/Abstract])) OR (Erlotinib[Title/Abstract])) OR (N-(3-ethynylphenyl)-6,7-bis(2-methoxyethoxy)quinazolin-4-amine[Title/Abstract])) OR (Tarceva[Title/Abstract])) AND (non-small cell lung cancer[MeSH Terms]) Filters: Clinical Trial

**b) Database: Embase (383)**

Search details were same to Pubmed.

**c) Database: The Cochrane Library (1217)**

#1 (erlotinib):ti,ab,kw OR (Hydrochloride, Erlotinib):ti,ab,kw OR (Erlotinib HCl):ti,ab,kw OR (HCl, Erlotinib):ti,ab,kw OR (OSI-774):ti,ab,kw (Word variations have been searched)

#2 (OSI 774):ti,ab,kw OR (OSI774):ti,ab,kw OR (CP 358774):ti,ab,kw OR (358774, CP):ti,ab,kw OR (CP 358,774):ti,ab,kw (Word variations have been searched)

#3 (358,774,CP):ti,ab,kw OR (CP-358,774):ti,ab,kw OR (CP358,774):ti,ab,kw OR (CP-358774):ti,ab,kw OR (CP358774):ti,ab,kw (Word variations have been searched)

#4 (11C-erlotinib):ti,ab,kw OR (11C erlotinib):ti,ab,kw OR (Erlotinib):ti,ab,kw OR (N-(3-ethynylphenyl)-6,7-bis(2-methoxyethoxy)quinazolin-4-amine):ti,ab,kw OR (Tarceva):ti,ab,kw (Word variations have been searched)

#5 #1 or #2 or #3 or #4

#6 (non-small cell lung cancer):ti,ab,kw

#7 #5 and #6

**C. Afatinib**

**a) Database: PubMed (81)**

Search details: search(((((((((((((afatinib[MeSH Terms]) OR ((2E)-N-(4-(3-Chloro-4-fluoroanilino)-7-(((3S)-oxolan-3-yl)oxy)quinoxazolin-6-yl)-4-(dimethylamino)but-2-enamide[Title/Abstract])) OR (BIBW-2992-MA2[Title/Abstract])) OR (BIBW 2992 MA2[Title/Abstract])) OR (BIBW-2992MA2[Title/Abstract])) OR (BIBW 2992MA2[Title/Abstract])) OR (BIBW2992 MA2[Title/Abstract])) OR (Afatinib Maleate[Title/Abstract])) OR (BIBW 2992[Title/Abstract])) OR (BIBW2992[Title/Abstract])) OR (BIBW-2992[Title/Abstract])) OR (Gilotrif[Title/Abstract])) OR (Afatinib Dimaleate[Title/Abstract])) AND (non-small cell lung cancer[MeSH Terms]) Filters: Clinical Trial

**b) Database: Embase (92)**

Search details were same to Pubmed.

**c) Database: The Cochrane Library (323)**

#1 (afatinib):ti,ab,kw OR ((2E)-N-(4-(3-Chloro-4-fluoroanilino)-7-(((3S)-oxolan-3-yl)oxy)quinoxazolin-6-yl)-4-(dimethylamino)but-2-enamide):ti,ab,kw OR (BIBW-2992-MA2):ti,ab,kw OR (BIBW-2992MA2):ti,ab,kw OR (BIBW 2992 MA2):ti,ab,kw (Word variations have been searched)

#2 (BIBW-2992MA2):ti,ab,kw OR (BIBW2992 MA2):ti,ab,kw OR (Afatinib Maleate):ti,ab,kw OR (BIBW 2992):ti,ab,kw OR (BIBW2992):ti,ab,kw (Word variations have been searched)

#3 (BIBW-2992):ti,ab,kw OR (Gilotrif):ti,ab,kw OR (Afatinib Dimaleate):ti,ab,kw (Word variations have been searched)

#4 #1 or #2 or #3

#5 (non-small cell lung cancer):ti,ab,kw

#6 #4 and #5

**D. Osimertinib**

**a) Database: PubMed (13)**

Search details: search(((((((((((((osimertinib[MeSH Terms]) OR (N-(2-((2-(dimethylamino)ethyl)methylamino)-4-methoxy-5-((4-(1-methyl-1H-indol-3-yl)-2-pyrimidinyl)amino)phenyl)-2-propenamide[Title/Abstract])) OR (mereletinib[Title/Abstract])) OR (osimertinib mesylate[Title/Abstract])) OR (osimertinib mesilate[Title/Abstract])) OR (mereletinib mesilate[Title/Abstract])) OR (N-(2-((2-(dimethylamino[Title/Abstract])ethyl)methylamino)-4-methoxy-5-((4-(1-methyl-1H-indol-3-yl)-2-pyrimidinyl)amino)phenyl)-2-propenamide methanesulfonate (1:1))) OR (AZD9291 mesylate[Title/Abstract])) OR (mereletinib mesylate[Title/Abstract])) OR (AZD-9291 mesylate[Title/Abstract])) OR (AZD9291[Title/Abstract])) OR (AZD-9291[Title/Abstract])) OR (Tagrisso[Title/Abstract])) AND (non-small cell lung cancer[MeSH Terms]) Filters: Clinical Trial

**b) Database: Embase (222)**

Search details were same to Pubmed.

**c) Database: The Cochrane Library (558)**

#1 (osimertinib):ti,ab,kw OR (N-(2-((2-(dimethylamino)ethyl)methylamino)-4-methoxy-5-((4-(1-methyl-1H-indol-3-yl)-2-pyrimidinyl)amino)phenyl)-2-propenamide):ti,ab,kw OR (mereletinib):ti,ab,kw OR (osimertinib mesylate):ti,ab,kw OR (osimertinib mesilate):ti,ab,kw (Word variations have been searched)

#2 (mereletinib mesilate):ti,ab,kw OR (N-(2-((2-(dimethylamino[Title/Abstract])ethyl)methylamino)-4-methoxy-5-((4-(1-methyl-1H-indol-3-yl)-2-pyrimidinyl)amino)phenyl)-2-propenamide methanesulfonate (1:1))):ti,ab,kw OR (AZD9291 mesylate):ti,ab,kw OR (mereletinib mesylate):ti,ab,kw OR (AZD-9291 mesylate):ti,ab,kw (Word variations have been searched)

#3 (AZD9291):ti,ab,kw OR (AZD-9291):ti,ab,kw OR (Tagrisso):ti,ab,kw (Word variations have been searched)

#4 #1 or #2 or #3

#5 (non-small cell lung cancer):ti,ab,kw

#6 #4 and #5

**E. Dacomitinib**

**a) Database: PubMed (3)**

Search details: search((((((dacomitinib[MeSH Terms]) OR (Vizimpro[Title/Abstract])) OR (N-(4-(3-chloro-4-fluoroanilino)-7-methoxy-6-quinazolinyl)-4-(1-piperidinyl)-2-butenamide[Title/Abstract])) OR (PF 00299804[Title/Abstract])) OR (PF00299804[Title/Abstract])) OR (PF-00299804[Title/Abstract])) AND (non-small cell lung cancer[MeSH Terms]) Filters: Clinical Trial

**b) Database: Embase (39)**

Search details were same to Pubmed.

**c) Database: The Cochrane Library (74)**

#1 (dacomitinib):ti,ab,kw OR (Vizimpro):ti,ab,kw OR (PF 00299804):ti,ab,kw OR (PF00299804):ti,ab,kw OR (PF-00299804):ti,ab,kw (Word variations have been searched)

#2 (N-(4-(3-chloro-4-fluoroanilino)-7-methoxy-6-quinazolinyl)-4-(1-piperidinyl)-2-butenamide):ti,ab,kw (Word variations have been searched)

#3 #1 or #2

#4 (non-small cell lung cancer):ti,ab,kw

#5 #3 and #4

**Figure: Evaluation of publications bias.**


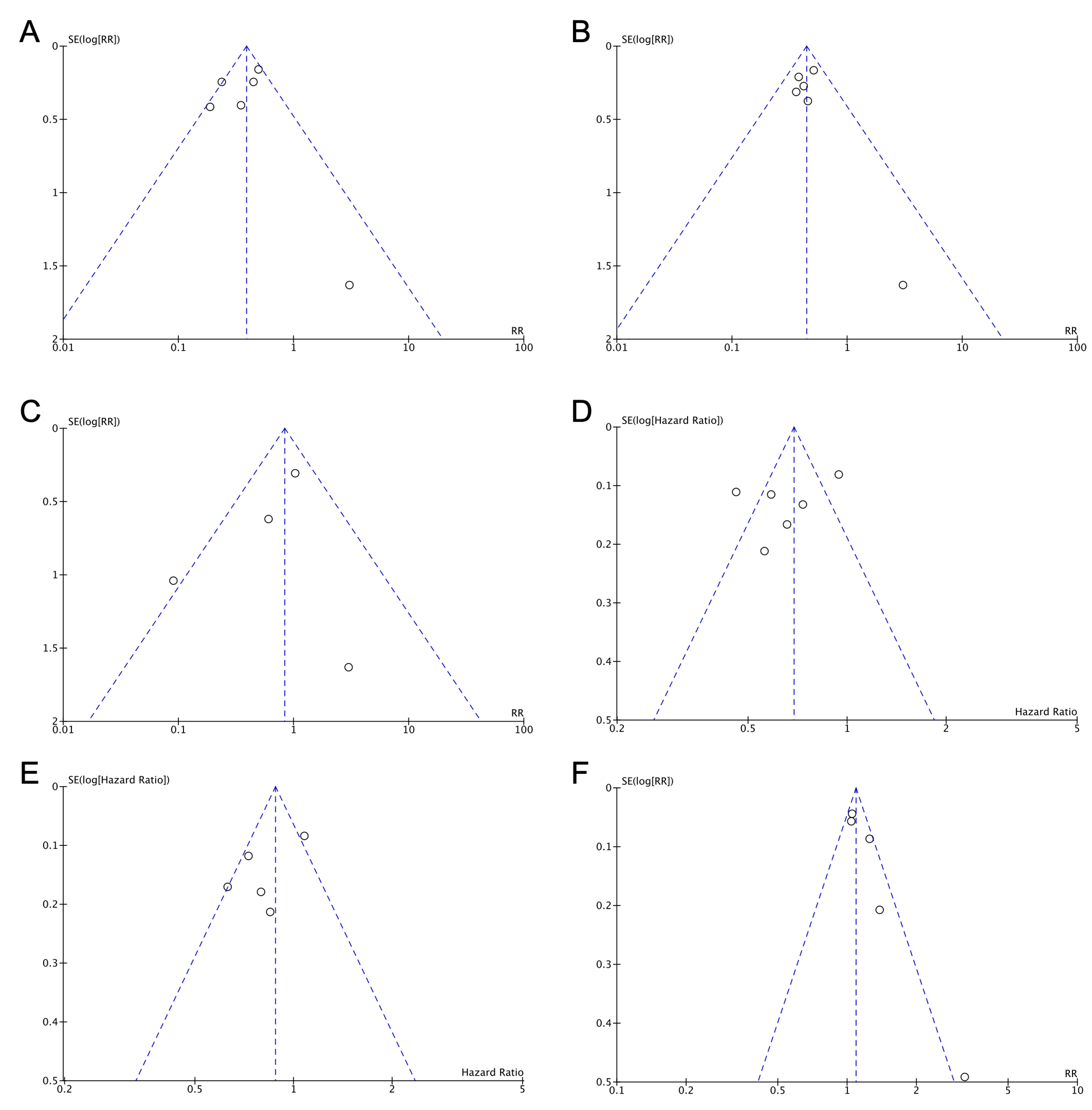


**Figure** Funnel plots of all grades ALT elevation (A), all grades AST elevation (B), all grades TB elevation (C), progression-free survival (D), overall survival (E) and overall response rate (F).
