# Supplementary material for: Hepatotoxicity and efficacy associated with first- and new-generation EGFR-TKIs in patients with NSCLC: a systematic review and meta-analysis
Source: BMC Cancer. 2025 Dec 29;25:1909. doi: 10.1186/s12885-025-15330-2 (PMC12751619; doi:10.1186/s12885-025-15330-2)
Supplement: Supplementary file 2 — Supplementary Material 2. [file 12885_2025_15330_MOESM2_ESM.docx]

**Highlights**

- New-generation TKI may decrease the risk of hepatotoxicity compared with gefitinib and erlotinib.
- New-generation TKI improve efficacy compared with gefitinib and erlotinib.
